# Flexible CNT-array double helices Strain Sensor with high stretchability for Motion Capture

**DOI:** 10.1038/srep15554

**Published:** 2015-11-04

**Authors:** Cheng Li, Ya-Long Cui, Gui-Li Tian, Yi Shu, Xue-Feng Wang, He Tian, Yi Yang, Fei Wei, Tian-Ling Ren

**Affiliations:** 1 Institute of Microelectronics and Tsinghua National laboratory for Information Science and Technology, Tsinghua University, Beijing 100084, China; 2 Beijing Key Laboratory of Green Reaction Engineering and Technology and Department of Chemical Engineering, Tsinghua University, Beijing 100084, China

## Abstract

Motion capture is attracting more and more attention due to its potential wide applications in various fields. However, traditional methods for motion capture still have weakness such as high cost and space consuming. Based on these considerations, a flexible, highly stretchable strain sensor with high gauge factor for motion capture is fabricated with carbon nanotube (CNT) array double helices as the main building block. Ascribed to the unique flexible double helical CNT-array matrix, the strain sensor is able to measure strain up to 410%, with low hysteresis. Moreover, a demonstration of using this strain sensor for capture hand motion and to control a mechanical hand in real time is also achieved. A model based on finite difference method is also made to help understand the mechanism of the strain sensors. Our work demonstrates that strain sensors can measure very large strain while maintaining high sensitivity, and the motion capture based on this strain sensor is expected to be less expensive, more convenient and accessible.

Strain sensors get increasing attention recently due to their wide applications in motion capture, respiratory monitoring, robot control and robotic skin, etc. Motion capture, especially, can be commonly found in surveillance, military, entertainment, sports and medical use etc^1^,^2^. With the rapid development of film industry, game industry and robot technologies, the demand for motion capture, as a crucial part of them, is growing rapidly. Currently, conventional human motion capture is mainly based on the several mechanisms as following: optical systems^3^,^4^, inertial sensors^5^,^6^, magnetic systems or mechanical systems^7^. Optical systems, which are intensively studied and widely used, normally have two categories: systems with markers and systems without markers. Marker systems require very complex equipment, special environment, and are expensive and space-consuming. However, markerless systems require further digital processing using complex algorithm and they are sensitive to environment but not as accurate as marker systems^4^. Inertial sensors are often limited by the volume of systems, while magnetic systems can be influenced by environment magnetic field. Mechanical system requires a mechanical structure to collect motion data, which is often uncomfortable. To reduce cost, shrink the volume and minimize the influence to performers while maintaining accuracy, it is highly desired to develop a new method for motion capture. Based on the fact that motion can cause large strain of skin, a possible approach is flexible strain sensors for capture motion.

Conventional strain sensors are mainly based on inflexible technologies^8^ using complex mechanical structures. Recently, flexible silicon strain sensor with large gauge factor has also been implemented^9^. However, although these sensors are suitable to measure strain precisely, the measurement range is much restricted by the materials. For instance, human motion can cause large strain of the skin^10^,^11^ (≥55%), which is hardly to be imitated by the routine strain sensors. Based on new material, strain sensors with relatively large measurement range have already been used in human motion detection^11^,^12^,^13^,^14^,^15^. However, to put strain sensors into practical use in motion capture, four aspects as following must be highly concerned: (1) capable of measuring large strain (≥55%); (2) stable enough to measure multiple deformations with low hysteresis; (3) large gauge factor in order to perceive strain precisely; (4) fast response to ensure fast signal acquisition.

Recently, strain sensors based on nanomaterial such as carbon nanotubes^11^,^16^,^17^ (CNTs), graphene^10^,^12^,^15^,^18^,^19^, nanoparticles^20^, nanowires^21^,^22^ have been reported. These sensors have advantages in some certain properties compared with conventional sensors. Some^10^,^17^,^18^,^21^,^22^ of these strain sensors have very large gauge factors, other works^11^,^12^,^16^,^20^ also report that strain sensors can measure large strain (≥80% strain). But they may not fulfill all the motion capture’s requirements simultaneously, such as large strain and large gauge factor. Moreover, stability is a crucial property of sensors. For example, repeatability and low hysteresis during loading and unloading. Strain sensors based on new nanomaterial have made improvements in some aspects, such as good repeatability, large measurement range, and gauge factor^15^, however, low hysteresis, fast and precise time response are still the outstanding challenge for practical and effective strain sensors .

Here we report a new type of resistive strain sensor based on a CNT-array double helice^23^ (CNTADH) film on thermoplastic elastomer (TPE) substrate which was able to endure a strain as high as 410%, with large gauge factor up to 12.1, fast response (time constant is ~0.5 s), high durability and repeatability. Most importantly, its hysteresis under 80% strain is 2% error on average. A wireless hand motion capture system is also made with these strain sensors, demonstration using this motion capture system to control a robotic hand in real-time is realized. The mechanism of the strain sensor is also investigated, a model based on finite difference method is made to explain the resistance change effect of the strain sensor. Simulation results conforms well with the experimental results, indicating that the mechanism used in the model where CNTADH film cracking into stripes is the main mechanism of the strain sensor.

## Results

### Device Fabrication

**Figure 1:**
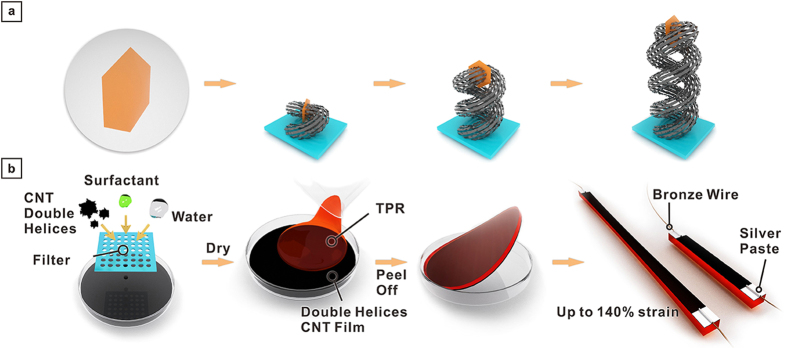
Growth of double CNT-array double helices and fabrication process of strain sensor. (**a**) Growth of CNT-array double helices. (**b**) Fabrication process of strain sensor based on CNT-array double helices.

Figure 1a illustrated the growth mechanism of the CNTADH with the lamellar layered double hydroxides (LDHs) as the catalyst precursor. Two CNT strands grew synchronously on both sides of one LDH flake and twisted to self-organize into a double helical structure under suitable space resistance^23^. Figure 1b shows the fabrication process of the CNTADH strain sensors. First, a sonicator is used to disperse the mixture of CNTADH, water and surfactant. The CNTADH dispersion was poured into a container and dried to obtain uniformed CNTADH thin film. The film is coated with TPE dissolved by toluene. TPE ,as substrate, is elastic and has large elongation (≥800%). The TPE solution was dried in ambient condition and removed from the container. During this process, TPE permeated in to CNTADH, so that the CNTADH thin film was lifted off with the TPE. Then the sample was cut and wired out using silver paste and copper wire.

### Material and Device Characteristics

**Figure 2:**
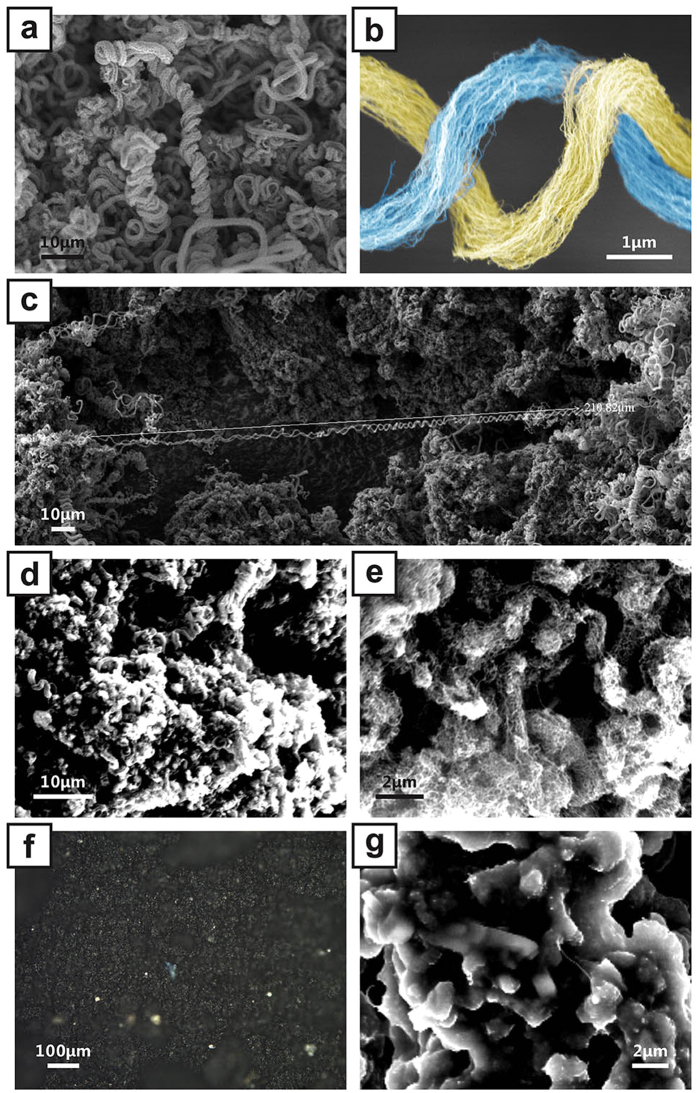
CNT-array double helices, CNT film and surface of the strain sensor. (**a**) Detail structure of CNT-array double helices in false color, indicating the two strands of CNT-array bundle. (**b**) A large amount of CNT-array double helices (**c**) A view of long CNT-array double helices. (**d**,**e**) SEM of the obtained CNT-array double helices thin film, after supersonic dispersion and dry. (**f**) Microscope photo of the strain sensor surface. (**g**) SEM of the strain sensor surface.

The over-all structure of a large amount of CNTADH is shown in Fig. 2a, indicating CNTADH is double helical and spring-like. Figure 2c indicates the double helical structure has good elasticity and can reach up to 210.82 μm. Figure 2b shows the detail structure of the CNTADH, showing the two strands formed by CNT array. SEM of CNTADH thin film, which is formed after sonication treatment and dried in the container, is also shown in Fig. 2d,e, indicating that the structure of CNTADH is well-preserved and is stable under short time sonicator treatment. Microscope image of strain sensor’s surface is shown in Fig. 2f, indicating the conductive surface consists of CNTADH is uniform after TPE coating. The sensor in Fig. 2f has been stretched to 200% strain over 5 times, the surface remains uniform, which also indicates the thin film is restorative. Figure 2g is SEM of strain sensor, shows that CNTADH film on sensor surface can maintain its structure after TPE coating.

### Test Results

**Figure 3:**
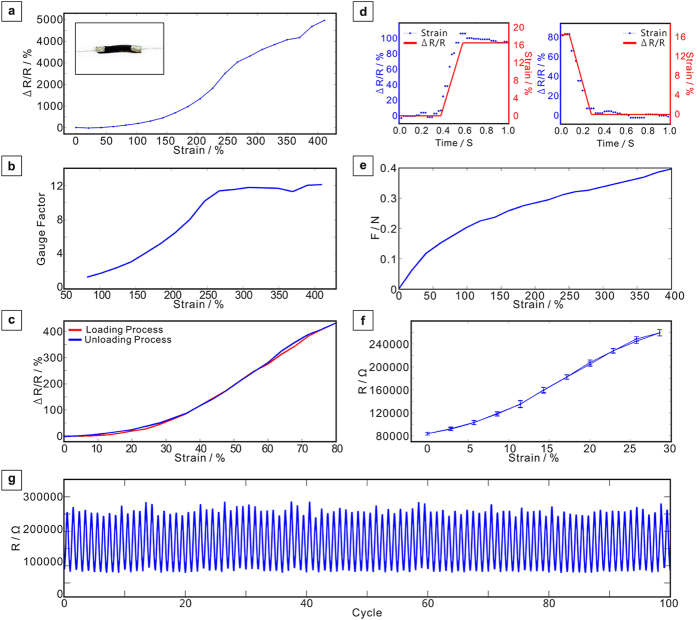
Test results of the strain sensors. (**a**) Relative resistance change versus strain. (**b**) Gauge factor versus strain. (**c**) Relative resistance change versus strain for loading and unloading cycle at 80% strain. (**d**) Time response of the strain sensor when applying a step expansion and step shrinkage. (**e**) The tension of the strain sensor versus strain. (**f**) 5 test cycles resistance change versus strain at 28.6% strain. Error bar indicates the deviation value of resistance. (**g**) The resistance versus 100 cycles of expansion and shrinkage.

Test results of our strain sensors are shown in Fig. 3. Relative resistance change versus strain is shown in Fig. 3a, indicating that the CNTADH strain sensor can measure strain up to 410%, and the resistance continuously increases while stretching. Electrical connectivity loses at larger strain, however strain sensor is still able to reverse to its initial state (Supplementary Fig. S1). At 250% strain, an inflection point can be observed in this curve. Moreover, resistance increases linearly after this point. Different samples and test results are also shown (Supplementary Fig. S2). Corresponding gauge factor during this process is also shown in Fig. 2b. During the stretching process, gauge factor increases to 12.1 when the strain is 410%. Our strain sensor’s measurement range is the largest among CNT based strain sensors^11^,^16^,^17^. Some other stretchable conductor can stand larger strain^24^,^25^, however their resistance does not change effectively with strain and cannot be used as strain sensors. Although a recent work^15^ on strain sensor shows larger measurement range and gauge factor, it didn’t concentrate on low hysteresis, and its time response has relatively larger glitches. Relative resistance change of loading and unloading process of 80% strain of the device is shown in Fig. 3c, which shows loading and unloading curves coincides well. The average error is less than 2%, showing little hysteresis under loading-unloading cycle. Time response of the sensor is shown in Fig. 3d, which shows the relative resistance change when applying a 0.2 s step change of strain (16.5%). Left figure shows the response when applying a step expansion, right figure corresponds to a step shrinkage, indicating the recovery time constant of our stain sensor is ~0.5 s, and shows little glitch. Tension of the strain sensor versus strain is also shown in Fig. 3f, overall tension is small when stretching, moreover it increase slower when strain is larger than 100%. This indicates the strain sensor using TPE as substrate may only cause little disturbance to measured objects. Figure 3f shows resistance change versus strain of 5 test cycles, at 28.6% strain. Deviation value of resistance is shown by error bar. Figure 3g shows the change of resistance versus cycle during 100 loading and unloading cycles of 28.6% strain, during this process the resistance shows high stability. It indicates the sensors have good stability during large amount of deformations. Short response time and low hysteresis is also important for motion capture. To our knowledge, no strain sensor based on CNT with better performance has been demonstrated when considering overall performance, including measurement range, gauge factor, stability, hysteresis, time response (Supplementary Table 1).

### Mechanism and Model

**Figure 4:**
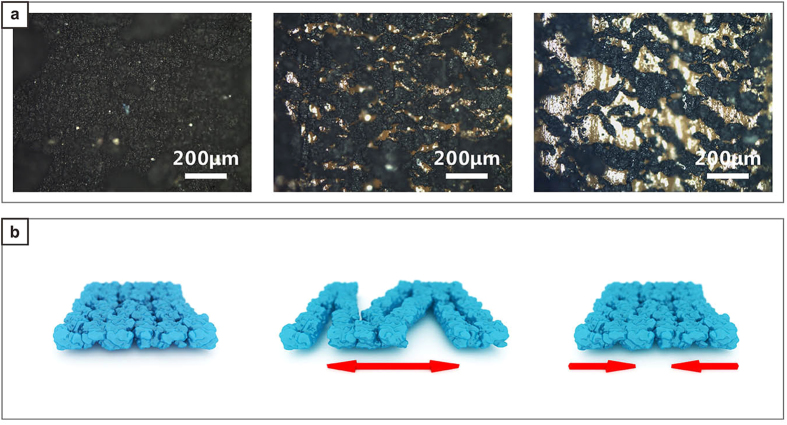
Microscope photo of the surface of strain sensor under different strain, sketch of the mechanism of the strain sensor. (**a**) Strain sensor under different strain, from the left strain become larger. (**b**) Schematic showing the mechanism of the strain sensor, from the left to right showing: the initial state, loading and unloading.

The CNTADH film, which forms the conductive surface of the strain sensor, under different strain is investigated under microscope, shown in Fig. 4a. Strain increases from left to right. Applied strain is in vertical direction. The pictures infer that when applying strain, the CNTADH film fractures into stripes, which forms the net-like conductive film. Figure 4b is a sketch showing the mechanism of the strain sensor. Blue part shows the CNTADH film. The fracturing tend to be perpendicular to the strain axis, which makes the conductive net more stretchable. CNTADH bundles tangles together, forms clusters of CNTADH bundles. When applying strain, some clusters have weaker connection to each other and fracture appears, the film forms a conductive net. CNT stripes’ width are ~20 μm, which is consistent with the SEM of the CNT stripe formed by CNTADH clusters (supplementary Figure S4), this validates the previous explanation. Larger strain can lead to smaller contact area between adjacent stripes, hence the resistance of the sensor increases. When strain decreases, adjacent stripes regain contact and resistance decreases. The inflection point found in the relative resistance change versus strain curve can attribute to the reposition of CNTADH cluster in the stripes when stretching, due to the properties of the substrate. Our sensors’ excellent performance can be explained by the combination of the unique properties of CNTADH and the substrate. First, Experiments show that CNTADH is capable of conducting large current^23^, which is suitable to make resistive strain sensors. Second, different from some other helical CNT^26^,^27^,^28^, whose diameters are under 50 nm and don’t have loose structure, CNTADH’s diameter is ~1 μm, which is relatively in larger scale and loose. Such feature can let TPE to soak in and make well contact, minimize the relative displacement of CNTADH and substrate, which lead to good stability and low hysteresis. Third, unlike other nanoparticles or nanowires like single wall CNT (SWCNT), carbon ponder, metal nano wires, CNTADH is spring like, which is suitable to sustain large strain. Fourth, conductive CNTADH film also have complex, bump and tangle structure, shown in Fig. 2g, this help to regain better contact between adjacent stripe after stretching and shrinking. Fifth, TPE can stand strain as much as 800%, which ensures substrate won’t restrict the measurement range.

Strain sensors based on CNT has been studied for years, however the mechanism of CNT strain sensors based on different type of CNT and different fabrication process varies a lot. Typically, CNT strain sensors can be sorted into two types, strain sensors based on CNT polymer composite and CNT thin film sensor. Models based on tunneling effect for CNT polymer composite based sensors has been made^29^. However tunneling effect causes resistance increases exponentially, which is different from our sensor. Moreover the microscope image, which is shown later, indicates the structural change of the CNTADH film is in micron scale. These shows that our sensor cannot be explained by tunneling effect. Simple model based on few resistances has also been made for strain sensors based on fracturing CNT thin film^11^. To understand the mechanism of our sensors more clearly and precisely, a model based on finite difference method has been made for the sensors (Supplementary Fig. S5). Based on the microscope characterization (Fig. 4a), we conjectured that the main mechanism is crack of the CNTADH film. This effect is analyzed in this model. The inflection point in the relative resistance change versus strain may due to some side effect induced by the test setup (Supplementary information 5), such as such as reposition of the CNTADH clusters caused by the nonuniform deformation of the sensor.

### Motion Capture Demonstration

**Figure 5:**
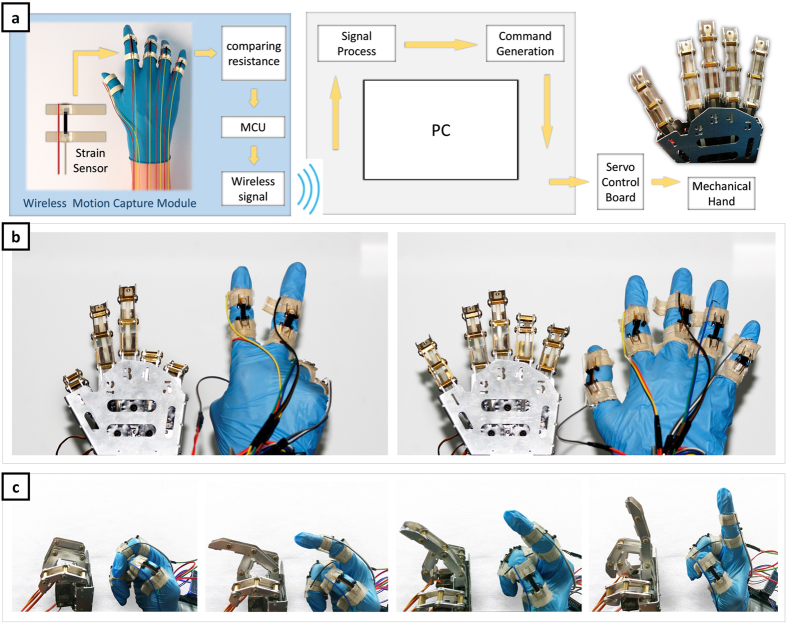
A demonstration of CNT-array strain sensor used in real time wireless hand motion capture and real time mechanical hand control. (**a**) Schematic of the system of motion capture and mechanical hand control. (**b**) Human hand gesture corresponding to mechanical hand gesture. (**c**) Mechanical hand reconstructing a finger’s position. From right to left finger bend gradually.

CNTADH strain sensor can satisfy motion capture’s requirements, here we show a demonstration of their usage in real time motion capture of human hand. The data collected from the sensors are also used to drive a mechanical hand and reconstruct gesture in real time. The schematic of the whole motion capture and mechanic hand control system is shown in Fig. 5a, it consists of three parts: wireless motion capture module, signal process and command generate system and mechanical hand. The strain sensors are attached to fingers and can sense the strain of fingers precisely. Real time hand motion capture is demonstrated, and are shown by controlling the mechanical hand. Mechanical hand reconstructing simple hand gesture is shown in Fig. 5b. Figure 5c shows a finger bending corresponding to the change of mechanical finger, which indicates mechanical hand can reconstruct one finger’s motion accurately. In Fig. 5c, our system shows good time response and high precision due to the good performance of the strain sensor, and is suitable for human motion capture. Moreover comparing to conventional motion capture, especially optical systems, this work has the following advantages: smaller room consumption; less sensitive to lighting conditions; it can capture performers’ motion at any place without any restrictions. This demonstration shows that CNTADH strain sensors may open up a new way for motion capture.

## Discussion

A high performance strain sensor based on CNTADH is made using a simple device fabrication method. The combination of CNTADH and substrate contributes to strain sensor’s excellent measurement range (410% strain). This strain sensor also has relatively large gauge factor, good time response, decent stability and little hysteresis. A model based on finite difference method is made and conforms well to experiment result, showing the main mechanism of our strain sensor is the restorable crack of the conductive layer. A demonstration using this strain sensor for hand motion capture and real time mechanical hand control is made. It shows that our sensors can be used in robot control, motion capture and may find broad application in wearable devices and wearable sensors. This work also showed a new possible way for motion capture, its advantages such as low-cost, low room-consumption may help it to be widely used in motion capture.

## Methods Section

### Strain sensor fabrication

CNTADH, surfactant and water are put into a test tube and treated by a sonicator for 20 minutes. Surfactant is from commercial detergent. The dispersion is filtrated by a commercial wire screen filter to remove large particles. Filtrated dispersion is poured into a petri dish and heated to 70 °C by hot plate. The substrate used here is thermoplastic elastomer (TPE) based on styrene ethylene butylene styrene copolymer (SBES), as obtained from commercial available TPE based toy. After the CNTADH dispersion is dried, TPE dissolved by toluene is coated on the CNTADH film. It is peeled off after dried in ambient condition for 72 hours. The round sample, shape defined by the petri dish, is then cut into stripes with 6 mm wide and ~3 cm length. Each side of the CNTADH film is connected to copper wire using silver paste.

### Strain Sensors testing

Relative resistance change was measured by mounting the strain sensor on to a micrometer caliper. Micrometer caliper can set the strain, resistance is measured by a digital multimeter DM3068 (RIGOL) and tension is measure by a force sensor. To measure time response, an operational amplifier (OPA) is used to convert the resistance in to potential signal, which is then measured by a virtual oscilloscope (INSTRUSTAR MDSO).

### Motion Capture System

Strain sensors are attached to a commercial available elastic band using tape (3 M VHB) and clips. Commercial available hook-and-loop fasteners are attached to the band, which is used to fasten the strain sensors on the fingers. The circuit board for wireless data transfer consists of a MCU (STC 89C52RC), comparing resistances and a wireless signal transfer module. Resistance signal of the strain sensors are transformed to potential signal using compare resistances. Wearable module consists of the circuit board and wrist band. Signal processing and command generation is performed on a PC using MATLAB. Signal is then transferred to a servo control board. Mechanical hand is controlled by five servos (MG996R), which are connected to the servo control board.

## Additional Information

**How to cite this article**: Li, C. *et al.* Flexible CNT-array double helices Strain Sensor with high stretchability for Motion Capture. *Sci. Rep.* **5**, 15554; doi: 10.1038/srep15554 (2015).

## Supplementary Material

Supplementary Information
