# Supplementary material for: Flexible CNT-array double helices Strain Sensor with high stretchability for Motion Capture
Source: Sci Rep. 2015 Nov 4;5:15554. doi: 10.1038/srep15554 (PMC4632108; doi:10.1038/srep15554)
Supplement: Supplementary Information [file srep15554-s1.pdf]

Flexible CNT-array double helices Strain Sensor with high stretchability for Wireless Motion Capture

—Supplementary Information

Cheng Li[1], Ya-Long Cui[1], Gui-Li Tian[2], Yi Shu[1], Xue-Feng Wang[1], He Tian[1], Yi Yang[1], Fei Wei[2], Tian-Ling Ren[1*]

[1]*Institute of Microelectronics and Tsinghua National laboratory for Information Science and Technology, Tsinghua University, Beijing 100084, China*

[2]*Beijing Key Laboratory of Green Reaction Engineering and Technology and Department of Chemical Engineering, Tsinghua University, Beijing 100084, (China)*

[*]Corresponding Author E-mail: RenTL@tsinghua.edu.cn

This file includes:

1. Measurement range test of the strain sensor
2. Different samples and their test results
3. Motion Capture System
4. SEM of the CNTADH stripe of the CNT film
5. Mechanism and Model.
6. Comparison with recent CNT based strain sensors

1. Measurement range test of the strain sensor

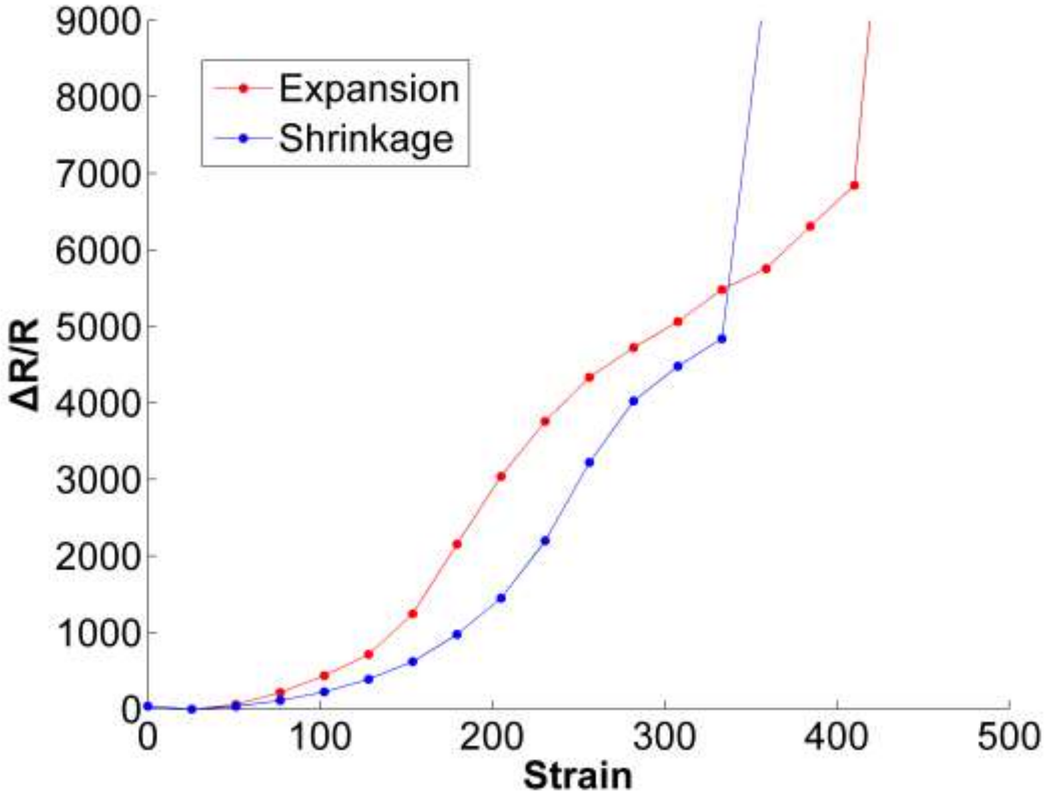

Figure S1. Measurement range test of the CNT array double helices strain sensor. Expansion process is shown with red curve. The strain sensor start to lose conductivity at around 330% strain, as resistance increases sharply. Blue dots indicates the shrinkage process. Even at this condition the strain sensor is able to reverse to its initial state, which shows decent stability.

2. Different samples and their test results

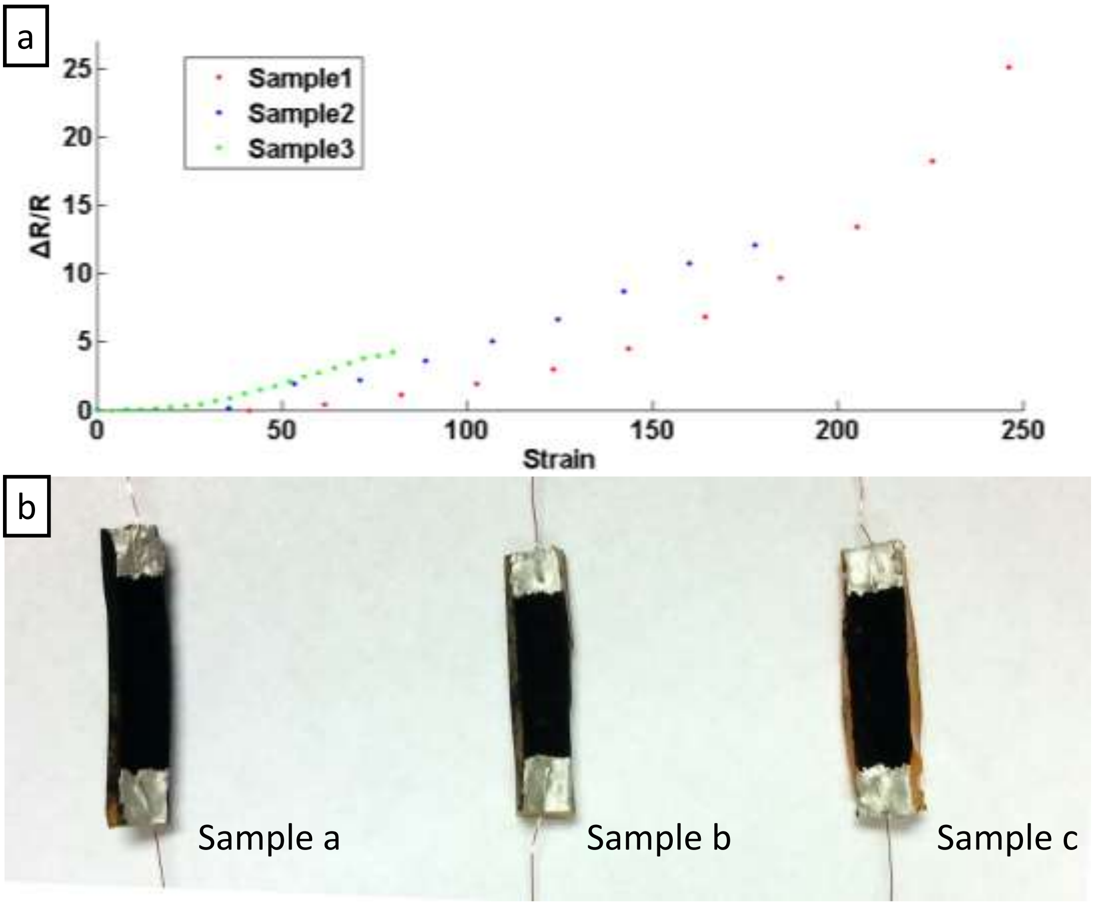

Figure S2. Different samples and their test results. (a) Relative resistance change versus strain of 3 different strain sensors. (b) A photo showing different strain sensors.

3. Motion Capture System

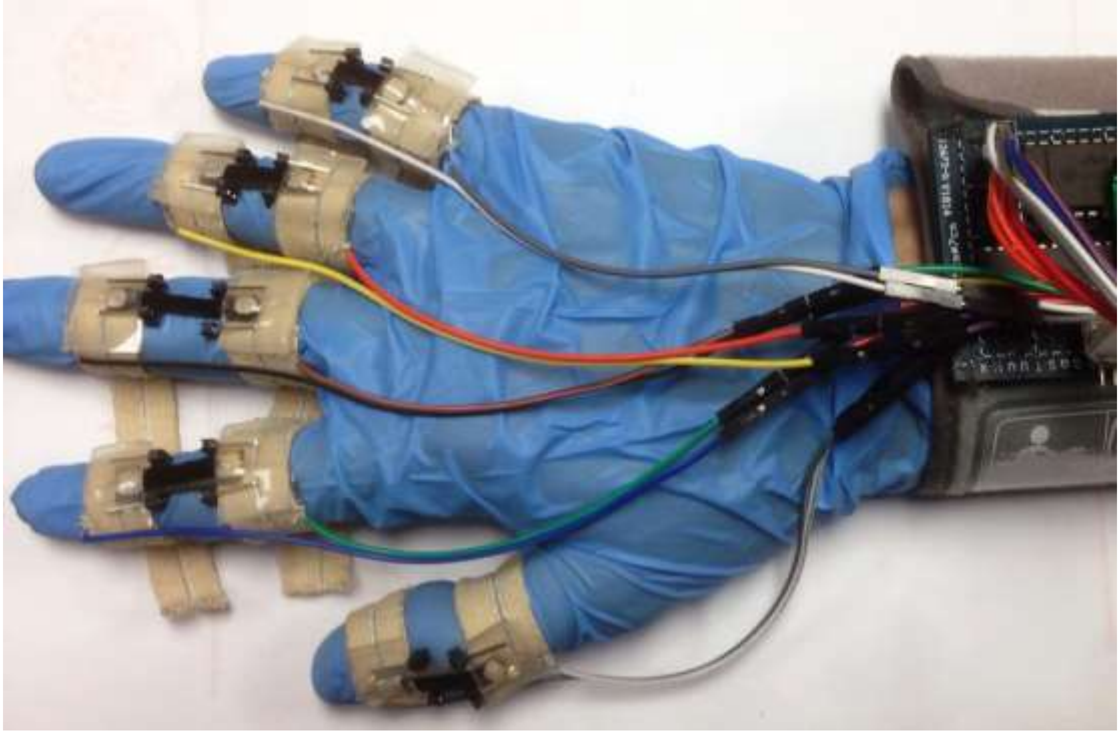

Figure S3. A view of the wireless motion capture system. Five strain sensors are attached to the fingers to perceive the motion of each finger. Wireless module on the wrist process the data from the strain sensors and transfer the signal through a Bluetooth module. Power used here is 5V DC input, which can be provided by a 9V battery and a voltage regulator. However to maintain stability, a DC power supply is still preferred.

4. SEM of the CNTADH stripe of the CNT film

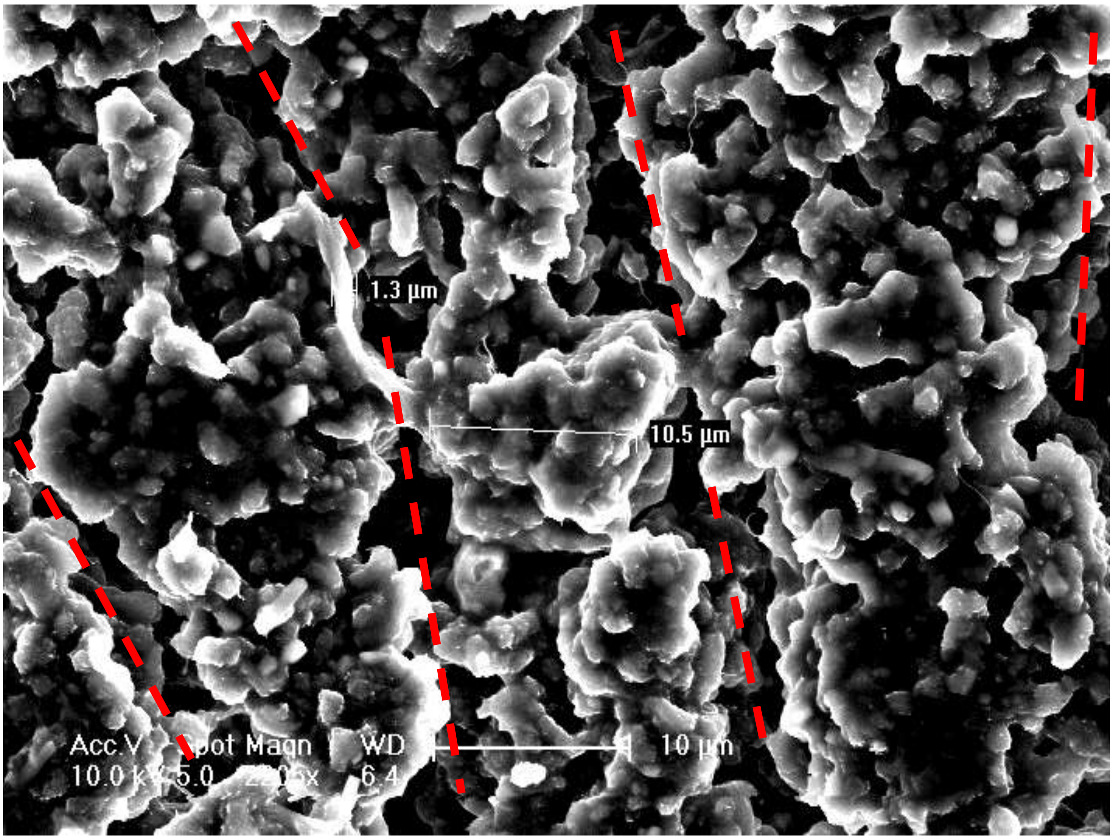

Figure S4. A view of the CNTADH stripe of the CNT film, form by CNTADH clusters. This figure is taken after the device has been stretched several times, shows that the CNTADH film fractures into stripes after strain applied. The stripe is formed by the CNTADH clusters and the width of the stripe is 10~20μm, this shows the same result as Figure 4a.

5. Mechanism and Model.

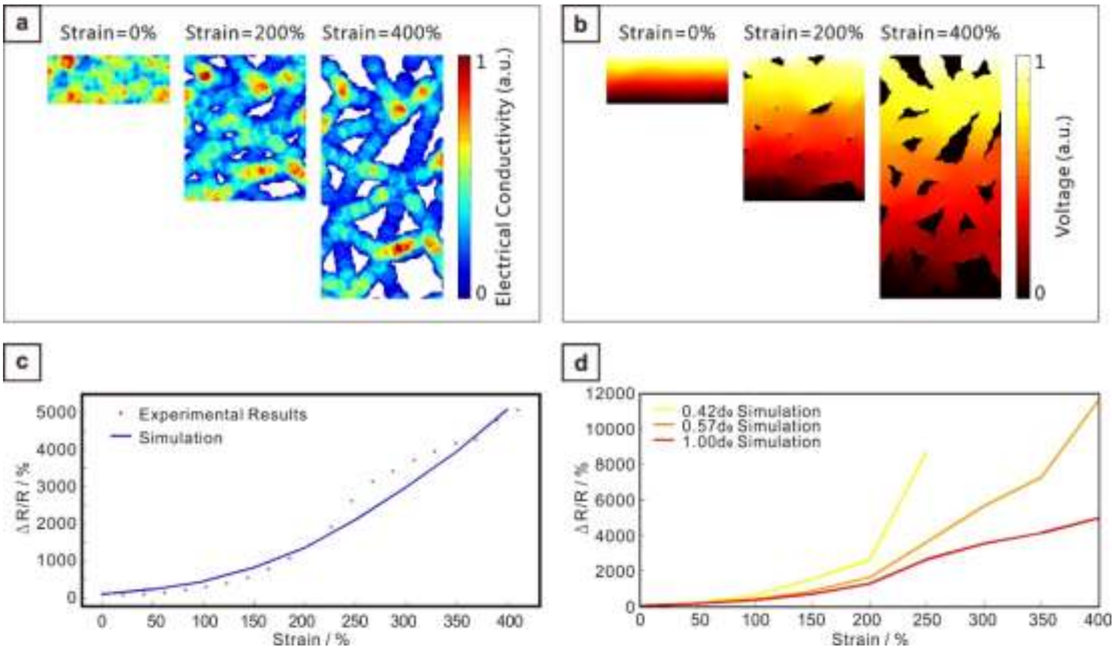

Figure S5. Simulation of the strain sensor. (a) Conductivity map of the strain sensor at different strain. (b) Electrical potential of the strain sensor at different strain. (c) Simulation results and the experiment results. (d) Simulation results with different CNT film thickness.

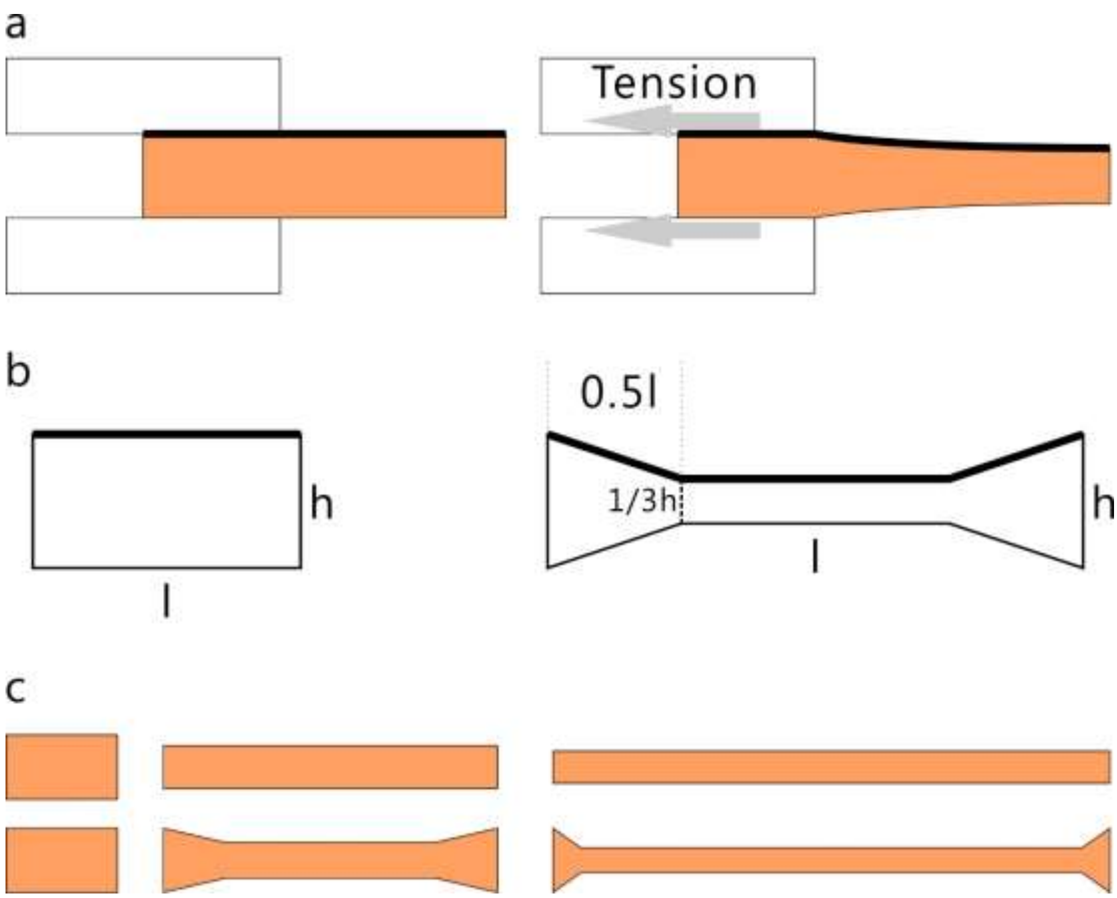

Figure S6. Nonuniform deformation of the sensor. (a) Vertical cross section of the sensor, showing how tension is applied. (b) Nonuniform deformation cause resistance increase. (c) Cross section of the sensor at different strain.

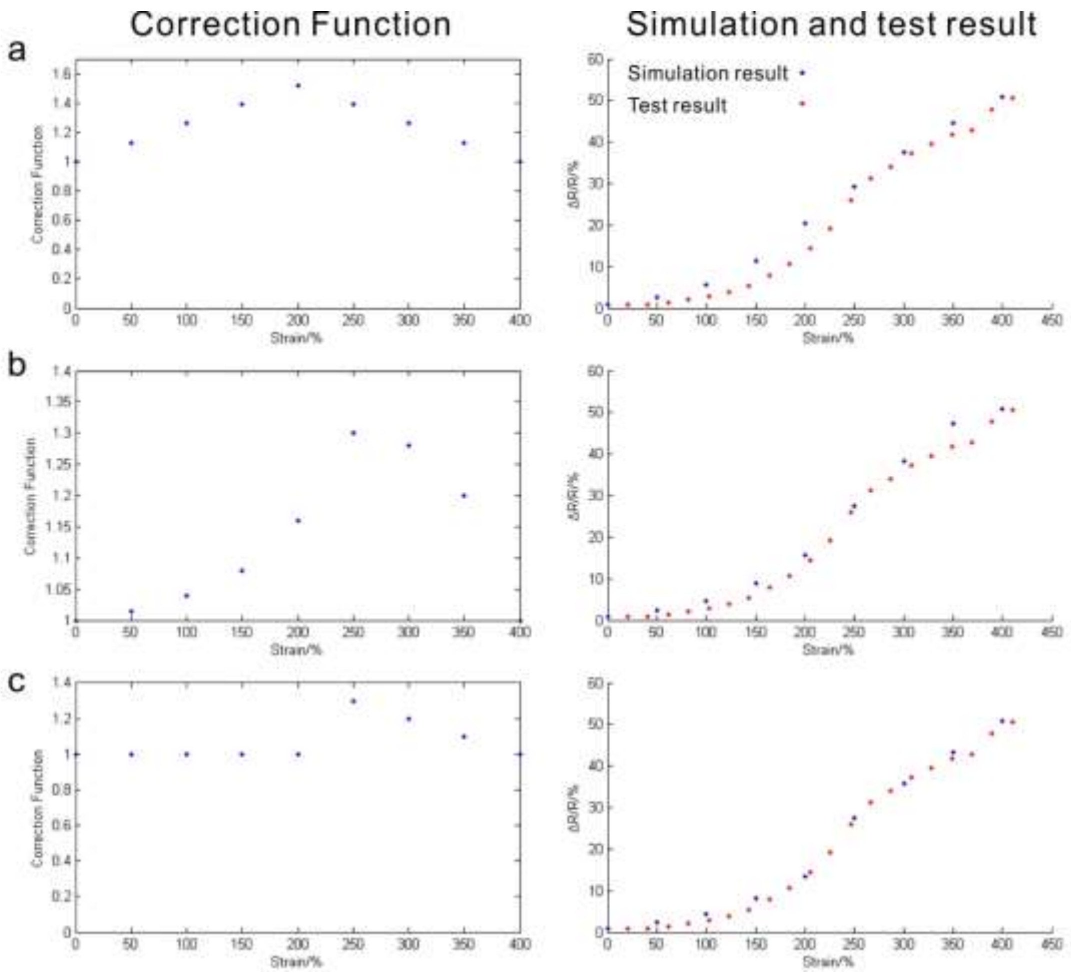

Figure S7. Different correction function and corresponding simulation result, comparing with test results.

To understand the mechanism of our sensors more clearly, a model based on finite difference method has been made for the sensors. The CNTADH film can be treated as randomly distributed clusters of CNTADH bundle, simulated by randomly placed conductive circular flake. To simulate the film crack, a random net is created, and the flakes move along the nearest net. Using a constant potential, resistance is calculated by R=V/I, where R is resistance, V is potential, I is current. Based on Ohm's Law:

$$J = \sigma E \tag{1}$$

Where J is current density, σ is electrical conductivity, E is electrical field, resistance can be calculated by finite difference method. The conductivity map at different strain is generated, shown in Figure S5a. Conductivity is proportional to the number of layers of CNT cluster. When strain is applied, the overlap area decreases and crack forms, hence the resistance increases. Figure S5b shows the potential map of the sensor, as strain increases, the electrical field decreases, which lead to smaller current and larger resistance. Simulated results and the experiment results are shown in Figure S5c.

Other side effects may cause the inflection point in the experiment results. One possible mechanism is the reposition of the CNTADH clusters. This is caused by the nonuniform deformation of the sensor caused by the set up. In an ideal situation, the strain sensor is stretched uniformly. However in the test set up, a clap structure is stick to the side of the strain sensor. When stretching, tension is applied to the side of the strain sensor, causing a necking effect (Figure S6a). This effect may lead to the result that the resistance of real device is larger than the ideal situation. A simple example is used to

show this effect (Figure S6b). The vertical cross section of effective part of the strain sensor is shown, although real condition is much more complex, this example can be used to explain why the necking of the sensor can cause the reposition of CNTADH and hence lead to resistance change from ideal condition. Assume the initial state is shown in the left in Figure S6b. When stretching, a simple model is shown in the right in Figure S6b. It is worth mentioning cross section of real device has a more complex shape. Since the thickness of the device is smaller than the width, necking in horizontal can be neglected. The area of the two cross section is the same:

$$\mathrm{hl} = \left(\mathrm{h} + \frac{1}{3}\mathrm{h}\right) * 0.5\mathrm{l} + \mathrm{l} * \frac{1}{3}\mathrm{h}$$

The CNTADH film is on top of the device, assume its initial thickness is t, width is w and length is l. The initial resistance of the device is:

$$\rho\frac{l}{wt}$$

ρ is the resistivity of thin film.

Resistance of the stretched state can also be calculated:

$$\int_0^{4l} \rho\frac{h * dx}{w * t * h'}$$

$$= 2 * \int_0^{l} \rho\frac{h * dx}{w * t * (1 - x * \frac{4l}{3})} + \rho\frac{3l}{wt}$$

$$= 4.648 * \rho\frac{l}{wt}$$

This is 1.162 times of ideal resistance. This factor is changing while strain increases, which infers that there is a correction function need to be multiplied to the original simulation result. To analyze why this effect can increase resistance, the sensor can be divided into 3 parts: transition part, stretched part, transition part. The ratio of the length

of transition part and the total length will decide how much the resistance increases. As the ratio become smaller, resistance increases less, this is because the sensor will have less difference with the original state. To show the basic trends of this mechanism, here we only analyze it qualitatively. When no strain is applied, ideal situation is the same as real situation. When strain is applied (Figure S6c middle), transition part, which is connecting the edge of the sensor and stretched part, is relatively large, comparing to the length of the whole device. When strain is very large (Figure S6c right), tension of tpe increase sharply, which will cause the stretched part less likely to be stretched. According to this, transition part become smaller. Thus the overall resistance tend to be similar to the simulation result. Relative length of the transition part determines the difference of real condition and simulation result. So when strain increase, correction function initially increases from 1, and then decreases.

Although the actual correction function cannot be obtained based simple analyze, it is useful to assume some basic function which is in agreement with the previous assumption as the correction function to show how this mechanism might influence the final result (Figure S7). These analyze still have limitations since they can only qualitatively explain the experiment results, further simulation may be done to improve the results.

$\Delta R/R$ versus strain of sensors with different thickness of CNTADH film are also simulated, shown in Figure S5d. It indicates that with thinner film, slope of the curve increases and the gauge factor is larger, but the measurement range will decrease.

Results show if the thickness of the sensors is 0.42 times of current sensor, the electrical connection completely breaks with strain less than 300%. Simulation also shows thicker CNTADH film has a smaller slope and smaller gauge factor. Since the thickness of the CNTADH film can be modulated by controlling the amount of CNTADH used when forming film, this shows using this method strain sensors with different gauge factor and different measurement range can also be made.

6. Comparison with recent CNT based strain sensors

**Work**	**Measurement Range**	**Gauge Factor**	**Hysteresis**
This device	410%	12.1	Average error <2%
1	0.002%	269	Not shown
2	~1.1%	~15	Not shown
3	~1.4%	~6	Not shown
4	150%	0.6	Average error >50%
5	280%	<0.82	Average error >50%

Table S1. Comparison of our device and some other CNT based strain sensor.

Properties of our device is compared with other CNT based devices, marked as 1[1], 2[2], 3[3], 4[4] and 5[5].

References

1. Chang, N.-K., Su, C.-C. & Chang, S.-H. Fabrication of single-walled carbon nanotube flexible strain sensors with high sensitivity. *Appl. Phys. Lett.* **92,** 063501 (2008).

2. Pham, G. T., Park, Y.-B., Liang, Z., Zhang, C. & Wang, B. Processing and modeling of conductive thermoplastic/carbon nanotube films for strain sensing. *Compos. Part B Eng.* **39,** 209–216 (2008).

3. Kang, I., Schulz, M. M. J., Kim, J. H. J., Shanov, V. & Shi, D. A carbon nanotube strain sensor for structural health monitoring. *Smart Mater. Struct.* **15,** 737–748 (2006).

4. Lipomi, D. J. *et al.* Skin-like pressure and strain sensors based on transparent elastic films of carbon nanotubes. *Nat. Nanotechnol.* **6,** 788–92 (2011).

5. Yamada, T. *et al.* A stretchable carbon nanotube strain sensor for human-motion detection. *Nat. Nanotechnol.* **6,** 296–301 (2011).
